# Differences in gas exchange, chlorophyll fluorescence, and modulated reflection of light at 820 nm between two rhododendron cultivars under aluminum stress conditions

**DOI:** 10.1371/journal.pone.0305133

**Published:** 2024-06-27

**Authors:** Jing Zhang, Yanxia Xu, Kaixing Lu, Zhengyu Gong, Zhenming Weng, Pengzhou Shu, Yujia Chen, Songheng Jin, Xueqin Li

**Affiliations:** 1 Jiyang College, Zhejiang A&F University, Zhuji, China; 2 Ningbo Key Laboratory of Agricultural Germplasm Resources Mining and Environmental Regulation, College of Science and Technology, Ningbo University, Ningbo, China; 3 Ecological Forestry Development Center of Suichang County, Suichang, China; United Arab Emirates University, UNITED ARAB EMIRATES

## Abstract

Aluminum (Al) toxicity is an important factor restricting the normal growth of plants in acidic soil. *Rhododendron* (Ericaceae) can grow relatively well in acidic soil. To uncover the adaptive mechanisms of photosynthesis under Al stress, the influence of Al stress on the photosynthetic activities of Al-sensitive (Baijinpao) and Al-resistant (Kangnaixin) rhododendron cultivars was examined by measuring gas exchange, chlorophyll fluorescence, and the modulated reflection of light at 820 nm. Under Al stress conditions, the net photosynthetic rate and stomatal conductance of the rhododendron leaves decreased, whereas the intercellular CO_2_ concentration increased. The Al stress treatment damaged the oxygen-evolving complex of the rhododendron seedlings, while also inhibiting electron transport on the photosystem II (PSII) donor side. In addition, the exposure to Al stress restricted the oxidation of plastocyanin (PC) and the photosystem I (PSI) reaction center (P_700_) and led to the re-reduction of PC^+^ and P_700_^+^. The comparison with Kangnaixin revealed an increase in the PSII connectivity in Baijinpao. Additionally, the donor-side electron transport efficiency was more inhibited and the overall activity of PSII, PSI, and the intersystem electron transport chain decreased more extensively in Baijinpao than in Kangnaixin. On the basis of the study findings, we concluded that Al stress adversely affects photosynthesis in rhododendron seedlings by significantly decreasing the activity of PSII and PSI. Under Al stress, Kangnaixin showed stronger tolerance compared with Baijinpao.

## Introduction

Aluminum (Al) accounts for approximately 8% of the total metal content, which is one of the most abundant metal elements in the crust [1]. Al in soil exists as part of insoluble silicates or Al trioxide, which are generally harmless to plants. Soil acidification (pH < 5.5) increases the solubility of Al and converts it to the trivalent cation form (Al^3+^), which is highly toxic to organisms [2] and is one of the major factors limiting plant growth and development [3–5]. Micromolar concentrations of Al in soil are sufficient for inducing irreversible toxic symptoms in plants, such as the rapid overproduction of reactive oxygen species, leading to oxidative bursts. The significant decrease in the uptake of water and nutrients due to Al stress can adversely affect plants [6].

Al stress is an important abiotic factor restricting the normal development of plants growing in acidic soil and has become a global problem [7, 8]. In terms of its global distribution, acidic soil (39.5 × 10^9^ hm^2^) primarily occurs in tropical, subtropical, and temperate regions. In China, acidic soil (total area of 2.03 × 10^7^ hm^2^) is distributed in 14 provinces and regions, but especially in the southwestern part of the country [6]. Al toxicity, which is a major factor disrupting the growth of plants in acidic soil, has hindered plant growth and agricultural development in areas with acidic soil [9]. In addition, the increase in the frequency of acid rain has accelerated soil acidification, resulting in the activation of a large amount of Al in the soil, which has severely restricted plant growth and development [6, 10].

Photosynthesis, which directly contributes to energy conversion and use, involves a series of complex biochemical reactions that are highly sensitive to metals [11]. The light-dependent reactions produce NADPH, ATP, and oxygen after light energy is absorbed by photosystems II (PSII) and I (PSI) and photosynthetic electron transport and photophosphorylation are initiated [12]. Of these two photosystems, PSII extracts electrons from water to reduce Q_A_, Q_B_, the plastoquinone (PQ) pool, the cytochrome b_6_f complex, and plastocyanin (PC), whereas PSI oxidizes the reduced PC, which reduces the electron acceptors on the acceptor side of PSI [13, 14]. Changes to any part of this process will alter the electron transport chain, thereby affecting photosynthetic efficiency [14]. The decrease in plant growth and metabolism caused by Al stress is associated with the inhibition of photosynthetic activities due to changes in stomatal conductance, ribulose-1,5-bisphosphate carboxylase/oxygenase activity, and chlorophyll contents [15–17]. Al stress can currently be discovered to dramatically lower the chlorophyll content and net photosynthetic rate (Pn) of plants in a wide variety of plant species, such as tobacco [18], barley [19], maize [20], soybean [21], citrus [22], eucalyptus [23], tea [24]. Al toxicity impaired PSII photochemical activity by blocking electron transport between Q_A_ and Q_B_, thus inhibiting photosynthesis in tobacco [18]. Reduced photosynthesis under Al stress is also associated with the inactivation of many chloroplast enzymes, which may be induced by oxidative stress [25]. High Al concentration increased the lipid peroxidation of soybean leaves, decreased cell membrane stability, changed the activity of superoxide dismutase (SOD), and then destroyed the system for detoxifying active oxygen species [21].

A technique that was recently developed for simultaneously measuring prompt chlorophyll fluorescence (PF), delayed chlorophyll fluorescence (DF), and modulated reflection of light at 820 nm (MR_820nm_) is convenient, flexible, and non-damaging to plants [26, 27]. When the dark reaction gives way to the light reaction, leaves release PF [14]. The subsequent reduction of electron acceptors on the acceptor side of PSII, in the PQ pool, and in the vicinity of PSI is linked to the redox state of the PSII reaction center (RC), which is dependent on PF [14]. When the light reaction gives way to the dark reaction, DF from leaves is produced [13]. This value reflects the recombination between the main electron acceptor Q_A_^−^ reduced by PSII in darkness and the oxidized donor (P_680_^+^) [28]. The MR_820nm_ provides information regarding the electron transport mediated by PQ and PSI receptors, thereby indicating the changes in the redox state of PSI RCs and PC [29]. Therefore, the redox reaction of PSI can be analyzed by measuring the light reflection at 820 nm [30, 31]. Concurrent measurements of PF, DF, and MR_820nm_ can yield complementary and parallel data regarding the composition or operation of the photosynthetic machinery [32]. Currently, the techniques have commonly been used in forestry [33], agriculture [34], horticulture [35], and other fields [36].

Rhododendrons are well-known economically valuable ornamental flowers. There are approximately 967 known species of rhododendrons worldwide, including approximately 562 species in China, which is considered to be the country with the richest wild rhododendron resources [37, 38]. In China, rhododendrons are mainly distributed in the southwestern region, in which soil acidification is severe [38]. Rhododendrons can grow well in acidic soil with high Al concentrations, implying they may have evolved complex mechanisms that enable them to effectively cope with Al stress [39]. Due to the different genetic backgrounds, the morphology, traditional use, phytochemistry and pharmacology of different varieties of rhododendrons are very different, which may lead to the great differences in their physiology and biochemistry [40]. In order to examine the alterations in the photosynthetic electron transport chain of rhododendron seedlings exposed to Al stress, we evaluated PF, DF, and MR_820nm_ concurrently in this work of Baijinpao and Kangnaixin, and revealed the changes of photochemical activity of different rhododendrons under Al stress.

## Materials and methods

### Plant materials and treatment

#### Experiment 1

The experimental materials were 21 rhododendron cultivars (S1 Table) from Jiashan United Agricultural Technology Co., Ltd. Rhododendron seedlings were transplanted in red soil (pH 4.48) collected from Gaoligong Mountain in Yunnan province, China and then grown in an artificial climate incubator under the conditions of 25°C 16-h day/18°C 8-h night, relative humidity of 70% (± 5%), and a photosynthetic photon flux density (PPFD) of 600 μmol m^−2^ s^−1^. The plants were cultured for 15 days before the Al stress treatment to ensure they were acclimated to the artificial climate incubator conditions and were growing normally. For these cultivars, the seedlings were treated using a completely random design, with six biological replicates. The 12 rhododendron seedlings of each species were randomly divided into two equal groups with 6 seedlings in each group. One group was treated twice a week with 200 ml 0.5 mM AlCl_3_ solution. The AlCl_3_ solution was in ultrapure water. The other group (control) was treated with same solution without AlCl_3_. All seedlings were watered as needed with tap water the rest of the time to prevent drought stress.

#### Experiment 2

*Rhododendron hybridum cv*. Kangnaixin and cv. Baijinpao were selected for further experiments. For both cultivars, the seedlings were treated using a completely random design, with six biological replicates. The 24 rhododendron seedlings of each species were randomly divided into two equal groups with 12 seedlings in each group. The remaining conditions were the same as those in the experiment 1.

### Determination of leaf gas exchange parameter

For the analysis of gas exchange parameters, leaves were chosen from each seedling starting from the first fully unfolded leaf from top to bottom. The Li-6400 XT portable photosynthetic measurement system (LI-COR, Lincoln, USA) with a fluorescence fluorometer (6400–40 leaf chamber) was utilized for this purpose. The CO_2_ concentration was set at 400 μmol mol^−1^, the chamber temperature was set to 25°C, the leaf area was set to 2cm^2^, and the PPFD and gas flow rate were set to 800 μmol mol^−1^ and 300 μmol m^−2^ s^−1^ respectively [22, 35]. On days 0, 7, 14, and 21 after the Al stress treatment, the determination was performed.

### Simultaneous measurement of PF, DF, and MR_820nm_

The Multifunctional Plant Efficiency Analyzer (M-PEA; Hansatech Instruments, Pentney, UK) was utilized for the simultaneous measurement of the leaf PF, DF, and MR_820nm_.

On days 0, 7, 14, and 21 after the Al stress treatment, leaves were treated in darkness for 30 min before the analysis. The actinic light LED provide homogeneous illumination with an intensity of 5000 μmol photons m^−2^ s^−1^ for 60s. MR_820_ nm was measured separately with far-red light of 1000μmol photons m^−2^ s^−1^. For the simultaneous measurements, the light–dark transition was completed at 300 μs after the exposure, with PF and MR_820nm_ signals recorded under light and then DF signals recorded in darkness. The wavelengths were 627±10 nm for the actinic light LED, 820±25 nm for the modulated light LED and 735±15 nm for the far-red light LED [14, 33]. Data were recorded with a variable rate: every 0.01ms from 0.01 to 0.3 ms, every 0.1ms from 0.3 to 3 ms, every 1 ms from 3 to 30 ms, every 10 ms from 30 to 300 ms, every 100 ms from 300 to 1000 ms [14, 33].

Fluorescence parameters were collected using the M-PEA. The parameters involved include the following: maximum quantum yield (at t = 0) for primary photochemistry (ϕ_Po_), quantum yield (at t = 0) of energy dissipation (ϕ_Do_), quantum yield (at t = 0) of electron transport (ϕ_Eo_), probability (at t = 0) that a trapped exciton moves an electron into the electron transport chain beyond Q_A_^-^(Ψ_Eo_), absorption flux (of antenna Chls) per RC (ABS/CS_m_), trapped energy flux (leading to Q_A_ reduction) per RC (TR_o_/CS_m_), electron transport flux (further than QA−) per RC (ET_o_/CS_m_), total energy dissipated per reaction center (RC) (DI_o_/CS_m_), the efficiency of an electron beyond that reduced PSI acceptors (δ_Ro_), performance index for energy conservation from exciton to the reduction of PSI end acceptors (PI_total_), performance index on an absorption basis (PI_ABS_).

### Statistical analysis

A two-factor analysis of variance (variety and Al stress treatment duration) was used. Using Duncan’s methods to analyze the significant difference among multiple sample groups. Data are herein provided as the average of six replicates ± standard error (SE). All data were processed and analyzed using SPSS 22.0 (IBM, Armonk, USA), with the default threshold for significance set at α = 0.05. The Origin 9.0 (Northampton, MA, USA) program was used for visualizing data.

## Results

**Fig 1:**
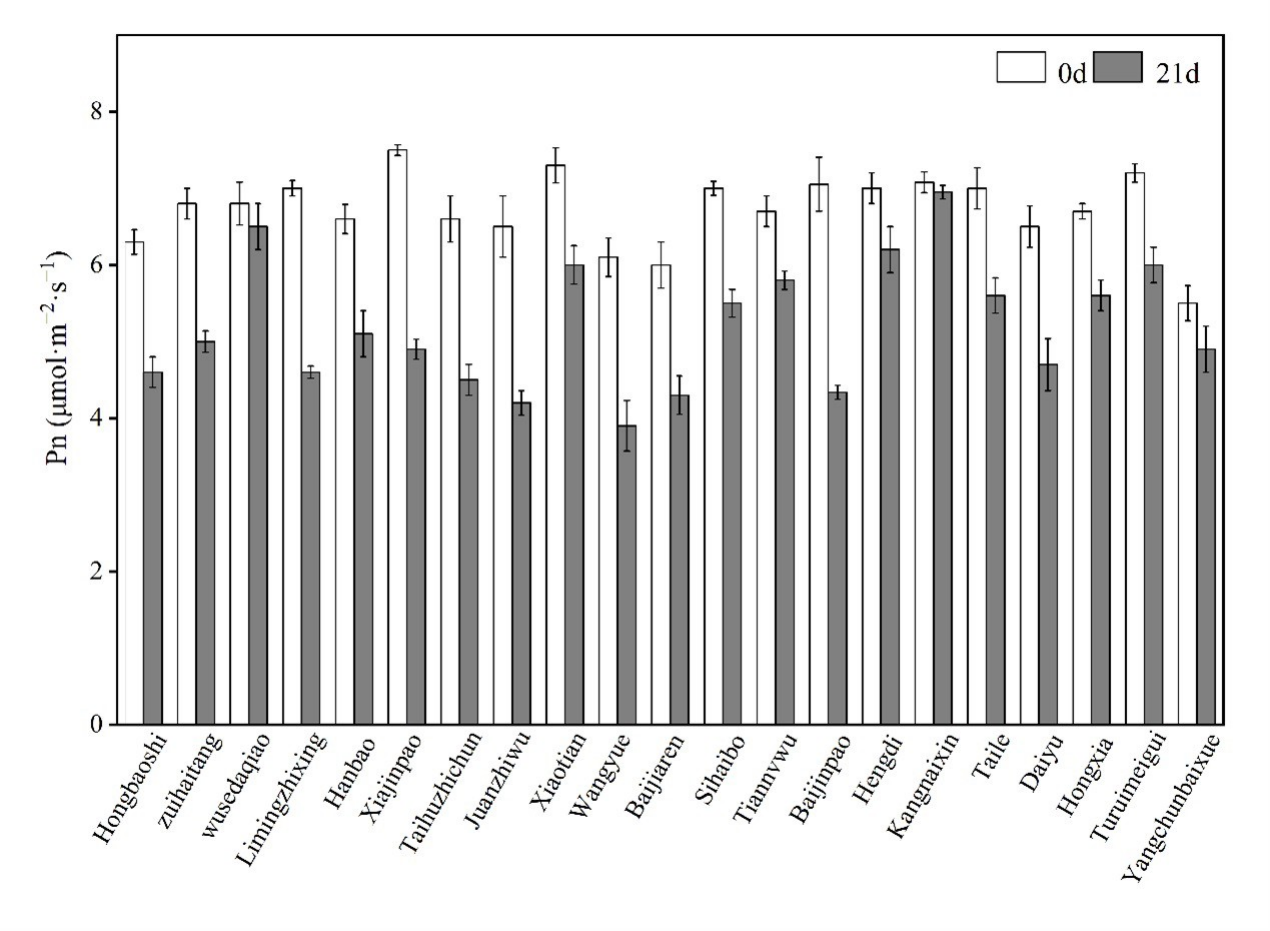
Variation of Pn of different rhododendron cultivars under Al stress.

After 21 days of Al stress, the net photosynthetic rate (Pn) of was decreased in 21 rhododendron cultivars (Fig 1). The lines Kangnaixin and Wusedaqiao were the most tolerant cultivar, while the lines Juanzhiwu, Wangyue and Baijinpao were the most sensitive cultivar. Based on this result, we selected Baijinpao as an Al-sensitive rhododendron and Kangnaixin as an Al-resistant rhododendron for subsequent experiments.

**Fig 2:**
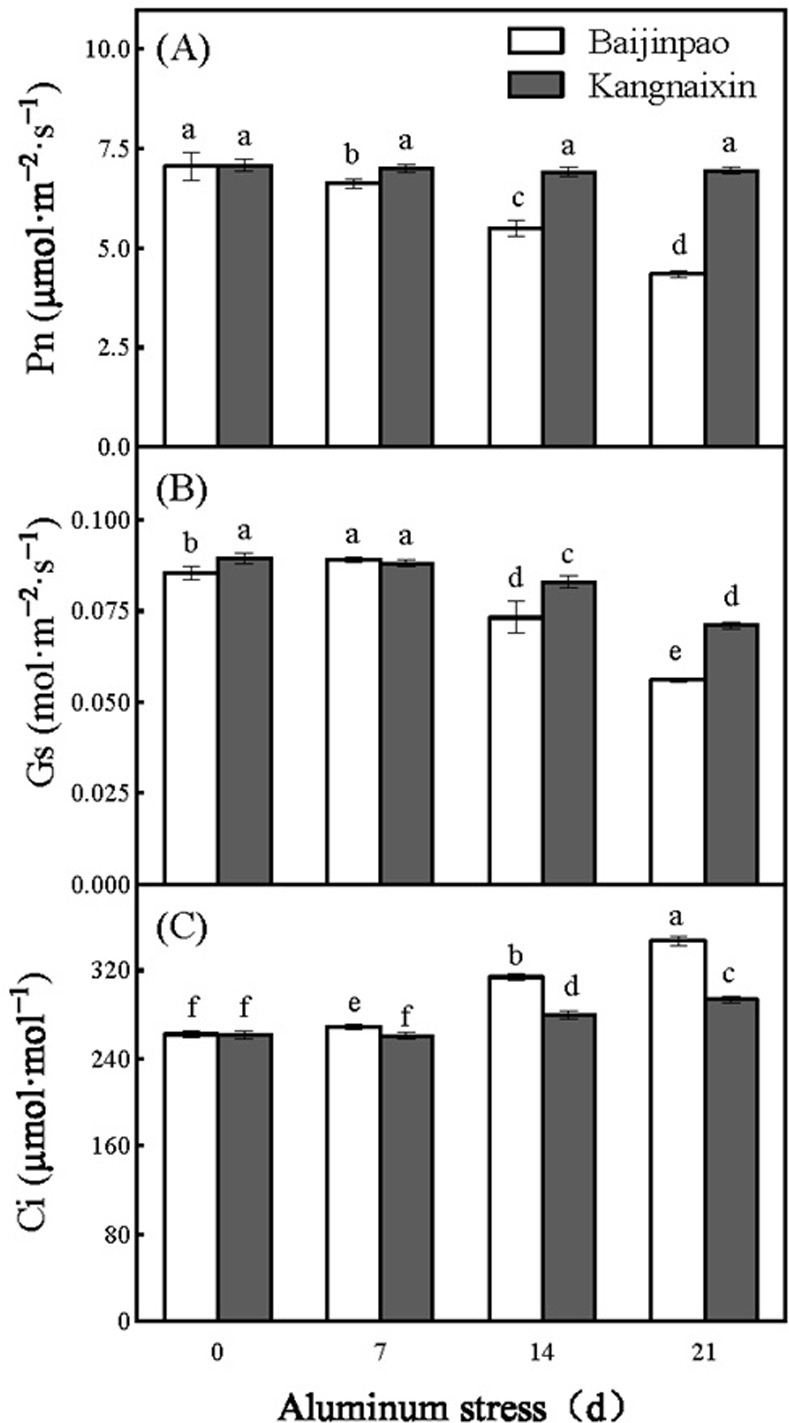
Variation in gas exchange parameters of leaves of Baijinpao and Kangnaixin under Al stress. (A). Pn, net photosynthetic rate; (B). Gs, stomatal conductance; (C). Ci, intercellular CO_2_ concentration. Duncan’s test was used to compare the changes between eight treatment combinations (two rhododendron cultivars × four Al treatment durations). Lowercase letters indicate P < 0.05.

After 21 days of stress, Pn decreased by 38.5% in Baijinpao, whereas it did not decrease significantly in Kangnaixin. In Baijinpao and Kangnaixin, stomatal conductance (Gs) decreased significantly (by 34.45% and 20.58%, respectively), whereas intercellular CO_2_ concentration (Ci) increased significantly (by 32.68% and 12.24%, respectively) (Fig 2).

**Fig 3:**
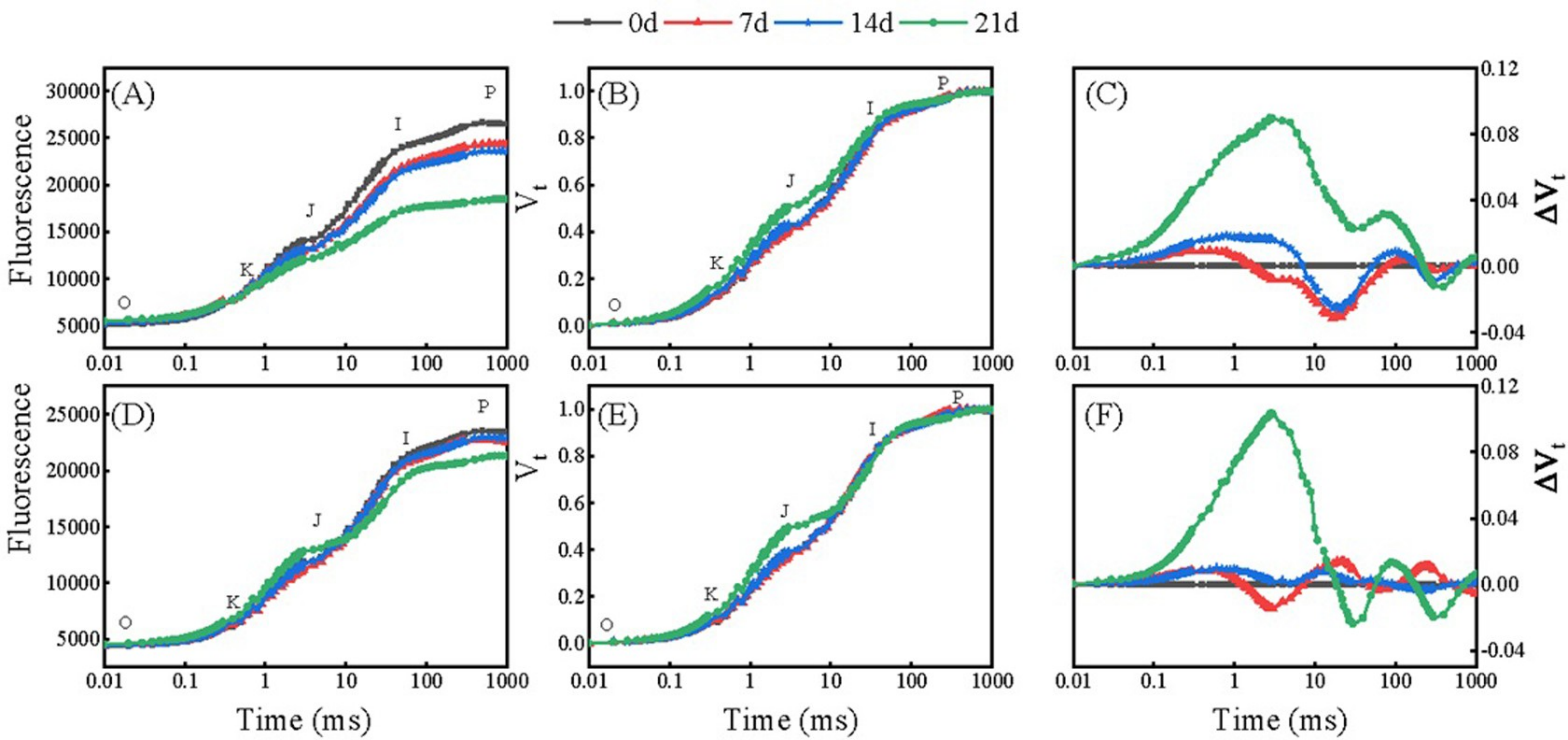
Changes in the OJIP curves of Baijinpao and Kangnaixin under Al stress conditions. (A–C): Baijinpao. (D–F): Kangnaixin. (A and D): OJIP curves for the Al stress treatment period. (B and E): V_op_ = (F_t_ − F_o_)/(F_m_ − F_o_). (C and F): ΔV_op_ = V_t_ (treatment) − V_op_ (control). F_o_: initial fluorescence intensity when all reaction centers are fully open; F_K_/ F_J_/ F_I_: fluorescence intensity at 300 μs/ 2 ms/ 30 ms; F_m_: maximum fluorescence intensity.

During the Al stress treatment, the fluorescence intensity of the rhododendron leaves initially increased and then decreased, with typical O, J, I, and P points and the OJIP transient (Fig 3A and 3D). As the treatment time increased, the I and P points of Kangnaixin gradually decreased, and more significantly decreases were detected in the O, J, I, and P points of Baijinpao (Fig 3A and 3D).

**Fig 4:**
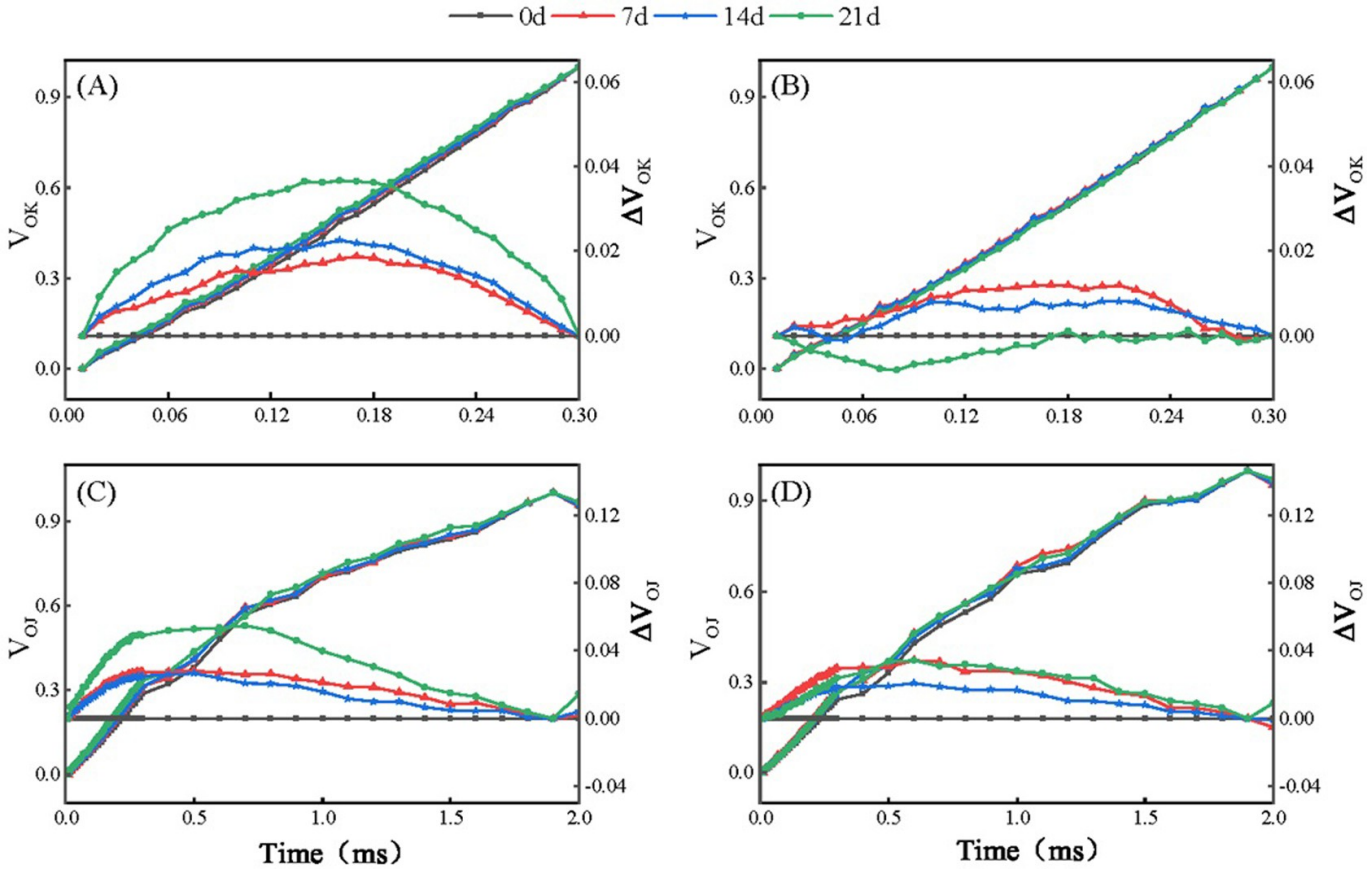
Changes in the L-band and K-band of Baijinpao (left) and Kangnaixin (right) under Al stress conditions. (A and B): V_OK_ = (F_t_ − F_o_)/(F_K_ − F_o_); ΔV_OK_ = V_OK_ (treatment) − V_OK_ (control). (C and D): V_OJ_ = (F_t_ − F_o_)/(F_J_ − F_o_); ΔV_OJ_ = V_OJ_ (treatment) − V_OJ_ (control).

**Fig 5:**
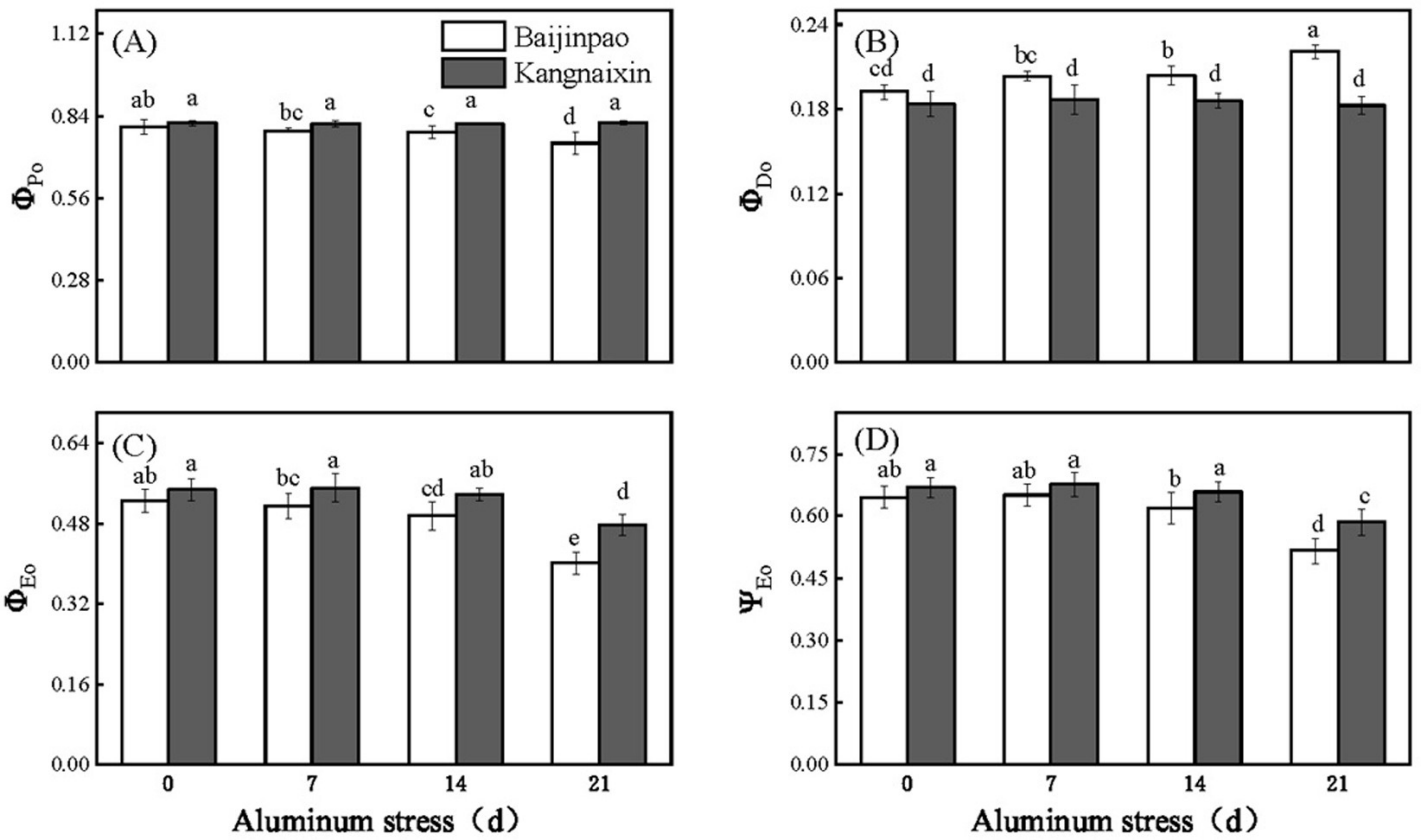
Variation in the energy distribution ratio of leaves of Baijinpao and Kangnaixin under Al stress. (A). ϕ_Po_: Maximum quantum yield for primary photochemistry; (B). ϕ_Do_: Quantum yield of energy dissipation; (C). ϕ_Eo_: Quantum yield of electron transport; (D). Ψ_Eo_: Probability that a trapped exciton moves an electron into the electron transport chain beyond Q_A_^-^. Duncan’s test was used to compare the changes between eight treatment combinations (two rhododendron cultivars × four Al treatment durations). Lowercase letters indicate P < 0.05.

**Fig 6:**
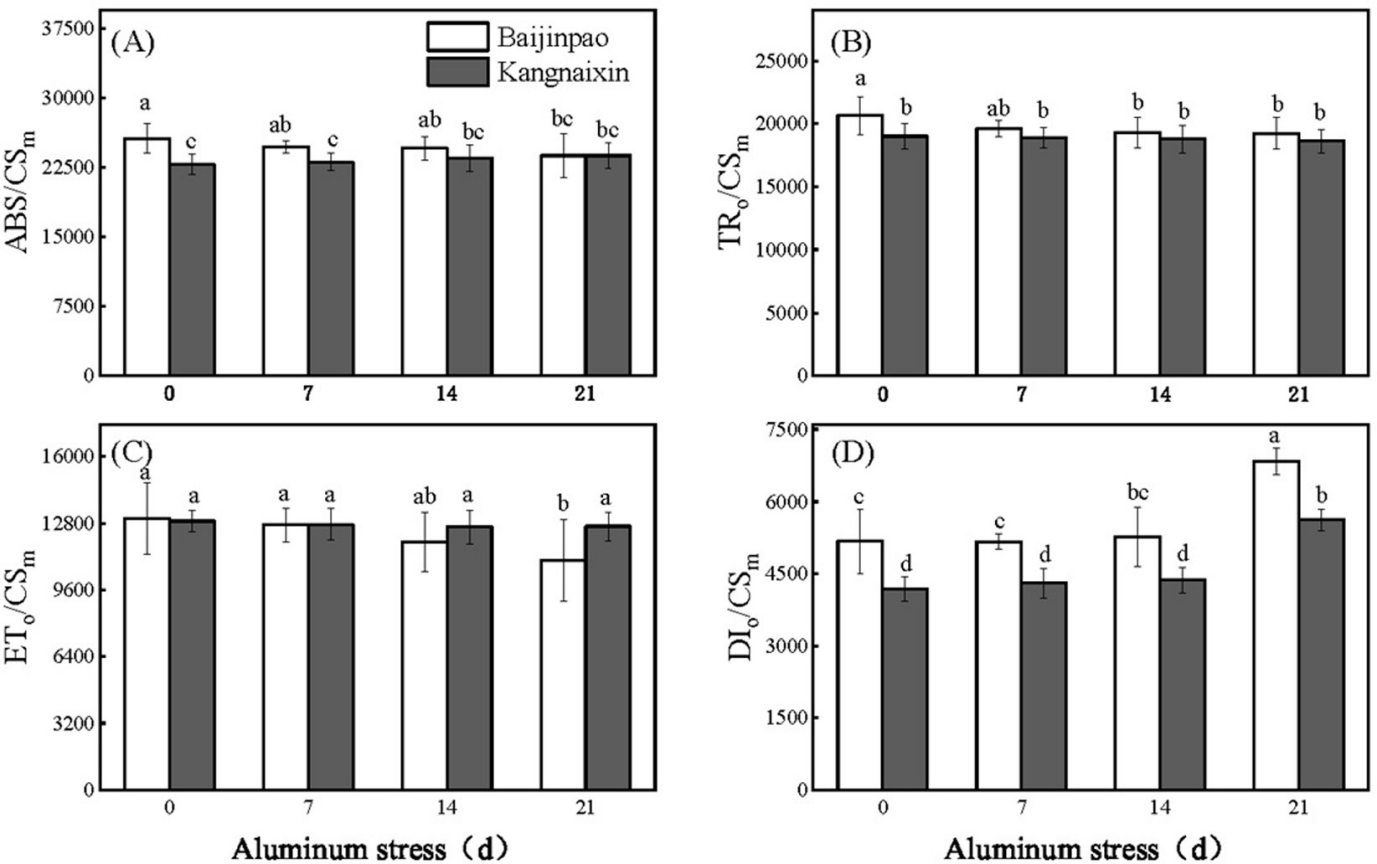
Variation in the energy fluxes per CS and the density of RCs of leaves of Baijinpao and Kangnaixin under Al stress. (A). ABS/CS_m_, absorption flux (of antenna Chls) per RC; (B). TR_o_/CS_m_: trapped energy flux (leading to Q_A_ reduction) per RC; (C). ET_o_/CS_m_: electron transport flux (further than Q_A_^−^) per RC; (D). DI_o_/CS_m_:,total energy dissipated per reaction center (RC). Duncan’s test was used to compare the changes between eight treatment combinations (two rhododendron cultivars × four Al treatment durations). Lowercase letters indicate P < 0.05.

**Fig 7:**
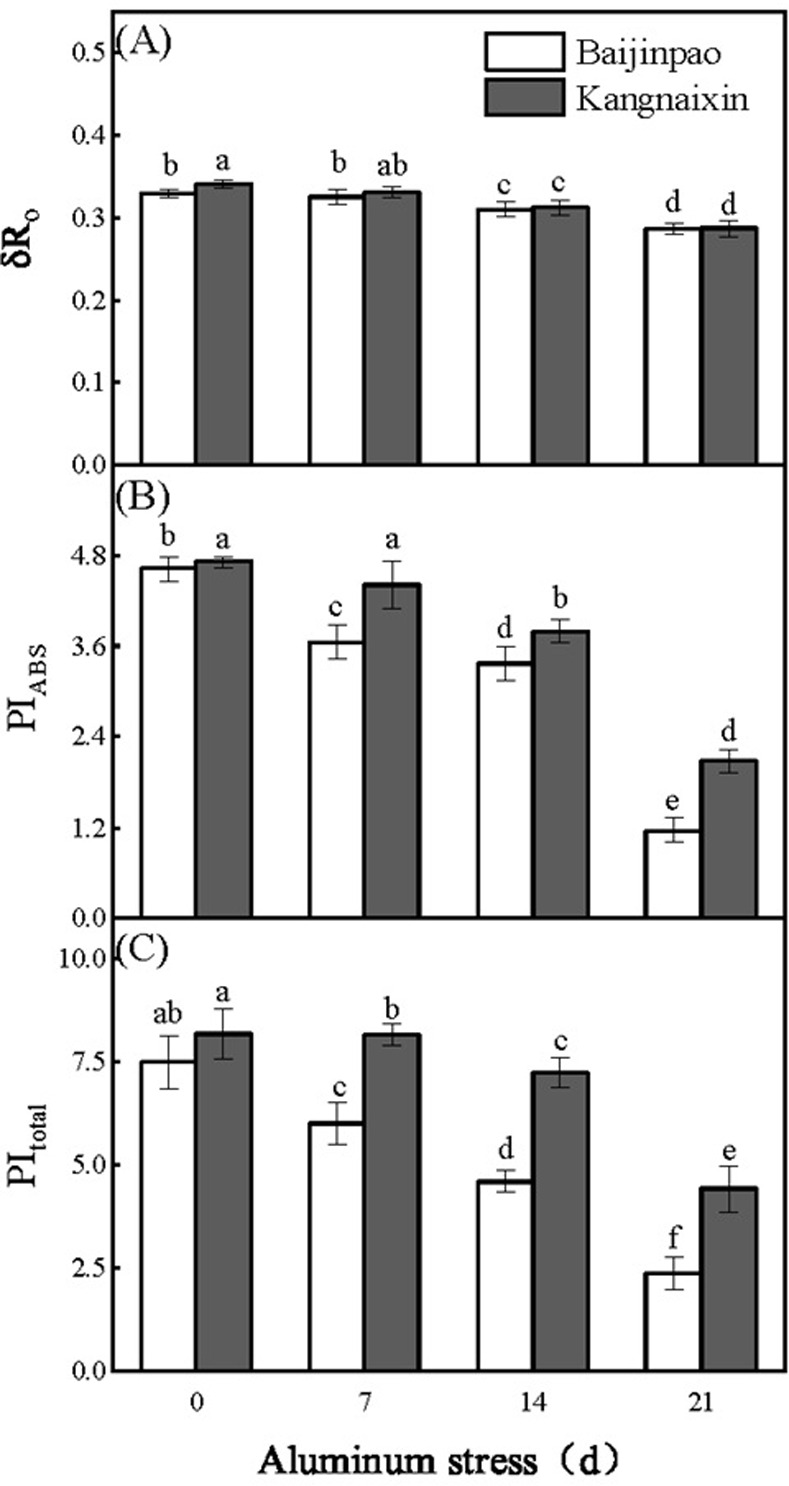
Variation in the photosynthetic performance of leaves of Baijinpao and Kangnaixin under Al stress. (A). δR_o_, the efficiency of an electron beyond that reduced PSI acceptors; (B). PI_total_, the performance index for energy conservation from exciton to the reduction of PSI end acceptors; (C). PI_ABS_: the performance index on an absorption basis. Duncan’s test was used to compare the changes between eight treatment combinations (two rhododendron cultivars × four Al treatment durations). Lowercase letters indicate P < 0.05.

After the OJIP data were normalized, the J, I, and P points of Baijinpao clearly increased, whereas obvious increases were detected for only the J and I points of Kangnaixin after 21 days of aluminum stress (Fig 3B and 3E). To facilitate the comparison, the OJIP curves were standardized (Fig 3C and 3F). The 21-day treatment of rhododendron seedlings with Al stress resulted in a significant increase in ΔV_t_ (Fig 3C and 3F). The O to J and O to K points of the OJIP curve were normalized and the differences were calculated to obtain the L-band (Fig 4A and 4B) and K-band (Fig 4C and 4D). After 21 days under Al stress conditions, the K-band increased significantly. The response of Baijinpao to Al stress was significantly greater than that of Kangnaixin (Fig 4C and 4D). The L-band of Baijinpao increased, while that of Kangnaixin decreased (Fig 4A and 4B). After 21 days of the Al stress treatment, ϕ_Po_, ϕ_Eo_, and Ψ_Eo_ of Baijinpao decreased by 6.9%, 23.7%, and 20.1%, respectively, whereas ϕ_Do_ increased by 14.8% (Fig 5). For Kangnaixin, ϕ_Po_ and ϕ_Do_ did not change significantly, but ϕ_Eo_ and Ψ_Eo_ decreased by 12.9% and 12.4%, respectively (Fig 5). Compared with the control levels (0d), after 21 days of Al stress, ABS/CS_m_, TR_o_/CS_m_, and ET_o_/CS_m_ of Baijinpao decreased by 7.2%, 6.7%, and 15.5%, respectively, but DI_o_/CS_m_ increased by 32.2% (Fig 6). For Kangnaixin, DI_o_/CS_m_ increased significantly by 32.2%, whereas others did not change significantly, (Fig 6). After the 21-day Al stress treatment, δR_o_, PI_total_, and PI_ABS_ decreased in Baijinpao (by 13.2%, 68.3%, and 74.8%, respectively) and Kangnaixin (by 15.7%, 45.9%, and 55.8%, respectively) (Fig 7).

**Fig 8:**
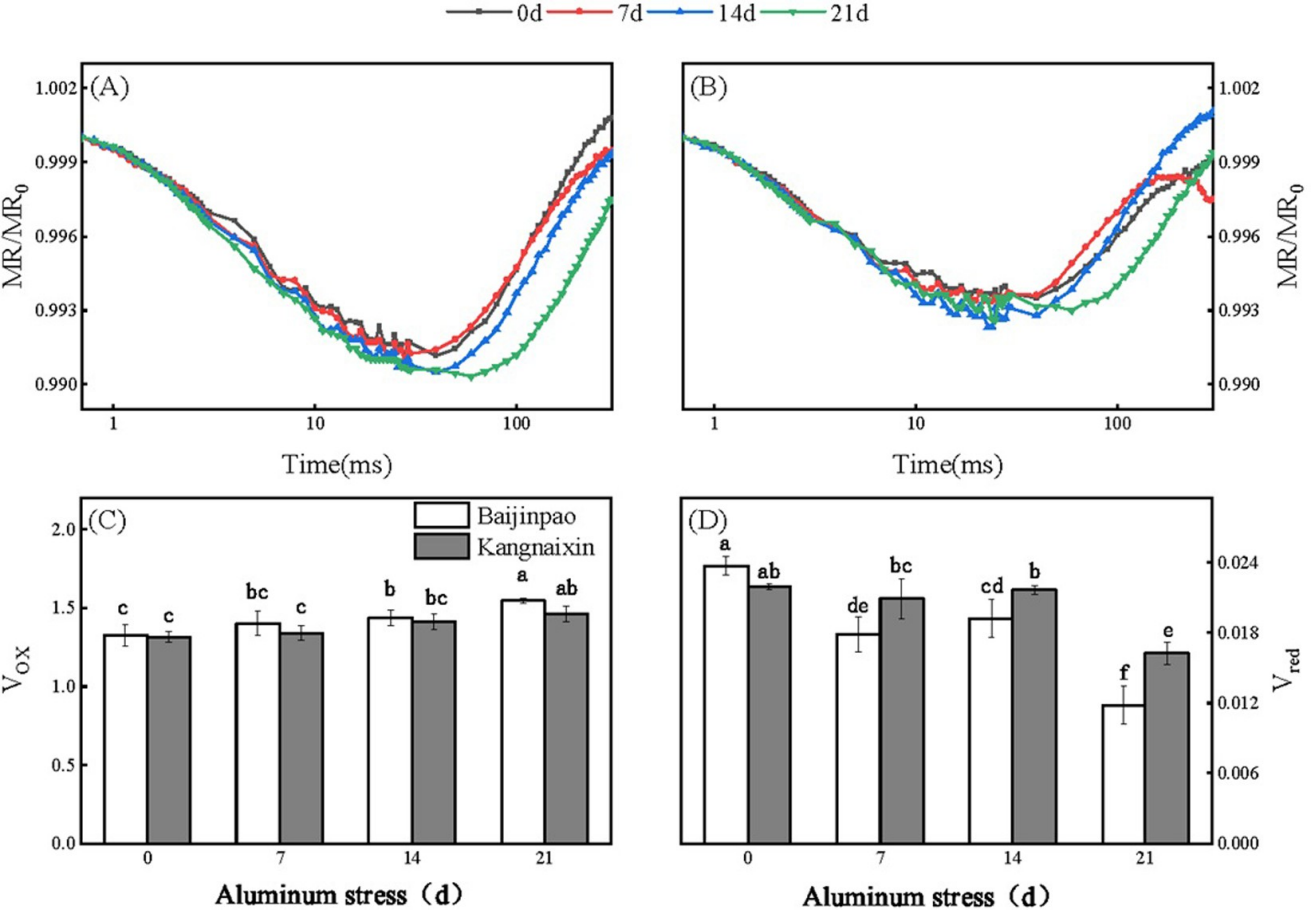
Changes in the MR_820nm_ curves and parameters of Baijinpao and Kangnaixin under Al stress conditions. (A/B): Effect of Al treatment on the MR_820nm_ curves of Baijinpao/Kangnaixin. (C): V_ox_, oxidation rate of PSI. (D): V_red_, PSI reduction rate. Duncan’s test was used to compare the changes between eight treatment combinations (two rhododendron cultivars × four Al treatment durations). Lowercase letters indicate P < 0.05.

At 21 days after the Al stress treatment, there were changes in the MR_820nm_ curve of the rhododendron, with a slight decrease in the lowest point (Fig 8A and 8B). As the treatment period progressed, the maximum slope of decrease in the MR/MR_0_ transient (V_ox_) increased, whereas the maximum slope of increase in the MR/MR_0_ transient (V_red_) decreased (Fig 8C and 8D). At 21 days after starting the Al stress treatment, V_ox_ increased by 16.8% and 11.2% for Baijinpao and Kangnaixin, respectively. Conversely, V_red_ decreased by 50.4% and 26% for Baijinpao and Kangnaixin, respectively (Fig 8C and 8D).

**Fig 9:**
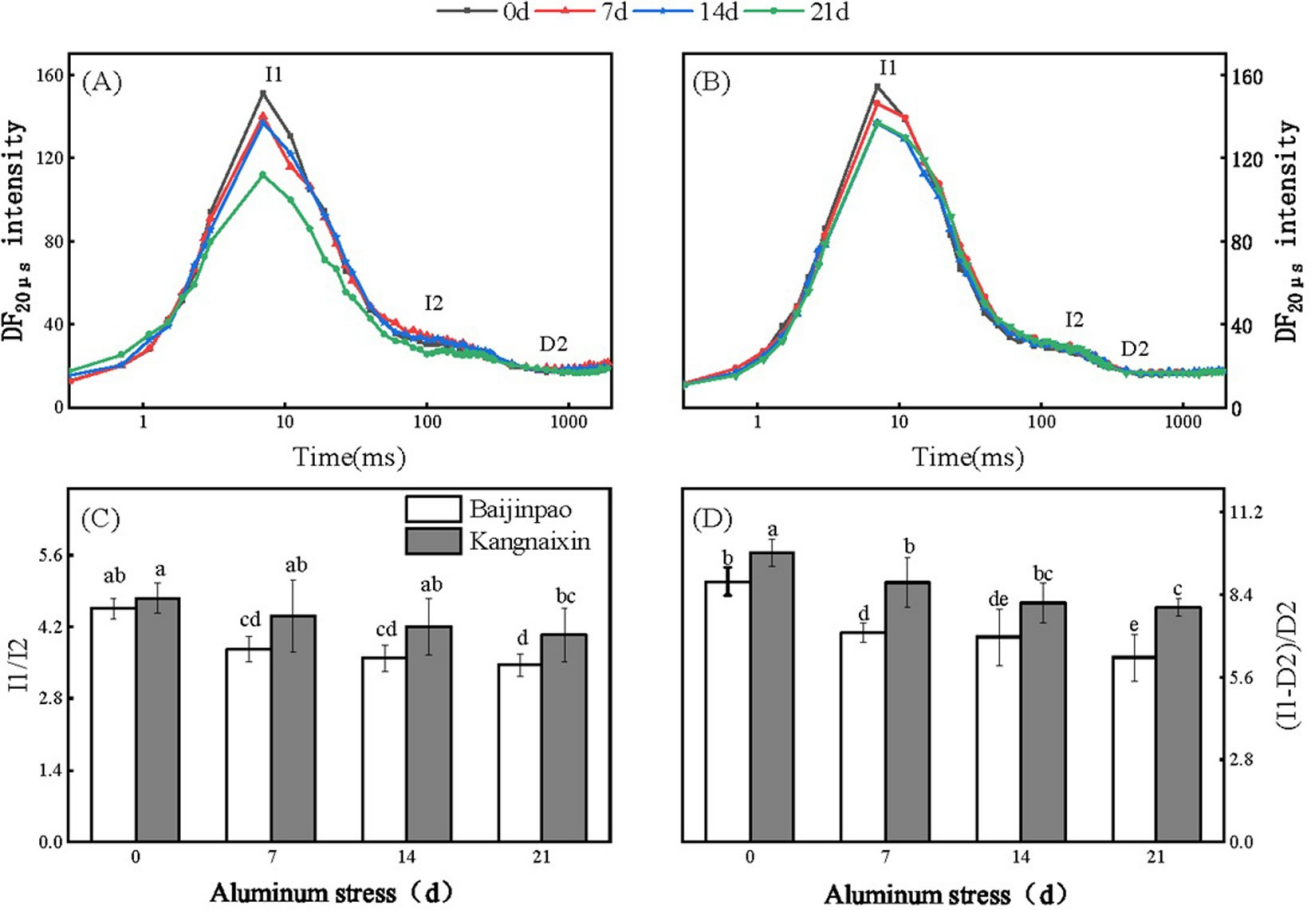
Effects of Al stress on the DF kinetics of Baijinpao and Kangnaixin. (A): Effects of Al stress on the DF curves of Baijinpao. (B): Effects of Al stress on the DF curves of Kangnaixin; I1: maximum value; I2: second peak value; D2: minimum value. Duncan’s test was used to compare the changes between eight treatment combinations (two rhododendron cultivars × four Al treatment durations). Lowercase letters indicate P < 0.05.

The construction of DF induction curves was based on the fluorescence signals measured at 20 μs (Fig 9A and 9B). Significant changes in the DF curves of the rhododendron seedlings were detected after 21 days of the Al stress treatment. There were significant decreases at the I1 point for Baijinpao and Kangnaixin, and the I2 point for Baijinpao also decreased (Fig 9A and 9B). Both I1/I2 and (I1 − D2)/D2 decreased significantly as the Al stress treatment progressed. After 21 days under Al stress conditions, I1/I2 and (I1 − D2)/D2 of Baijinpao decreased by 24.34% and 29.2%, respectively, which were larger than the corresponding decreases observed in Kangnaixin (by 15.15% and 19%, respectively) (Fig 9C and 9D).

## Discussion

In our study, Al stress treatment decreased Pn and Gs, but increased Ci, in both rhododendron cultivars (Fig 2), suggesting that the decline in Pn was caused by a non-stomatal factor [22, 41], which aligns with previous studies conducted on eucalyptus and citrus plants [23]. In addition, the Al stress-induced decrease in Pn was more significant in Baijinpao than in Kangnaixin, which indicated that Kangnaixin is more resistant to Al than Baijinpao.

When light irradiates the leaves of plants, the intensity of fluorescence emitted rises from the lowest point (O-point) to the highest point (P-point), which can be divided into three stages (O-J, J-I, and I-P) [42]. These phases can reflect the three different reduction processes in the electron transport chain [43]. There were obvious changes in the OJIP curve (Fig 3) as the Al stress treatment time increased, especially at 21 days, with the entire photosynthetic electron transport chain in the rhododendron seedlings damaged. After normalizing the OJIP data, we detected an increase of the J-point, but a decrease of the P-point, in both cultivars. Recent studies have demonstrated that an increase in the J-point may be due to a decrease in the electron transport efficiency at Q_A_, while a decrease in the P-point may be due to an increase in the non-radiative dissipation of PSII antenna pigments, a decrease in antenna pigment contents, a decrease in the PSII RC activities, damages to the PSI receptor side, or the denaturation and degradation of chlorophyll proteins [44]. In addition, an increase in the K-band may reflect the photoinhibition of PSII donors, which has been widely used as a specific indicator of the degradation of the oxygen-evolving complex (OEC) [45]. The increase of K band can be observed under many abiotic stresses [46, 47]. In the current study, the K-band increased during the Al stress treatment of both rhododendron cultivars, indicative of the Al stress induced OEC damage of PSII, ultimately resulting in decreased electron transport on the donor side of PSII. The increase of K-band was greater for Baijinpao than for Kangnaixin, indicating the donor-side electron transfer was weaker in Baijinpao than in Kangnaixin. The L-band reflects the energy connectivity among PSII units or between the antenna and the PSII RCs, with an increase in the connectivity indicative of an increase in damage [48, 49]. At 21 days after starting the Al stress treatment, the L-band of Baijinpao and Kangnaixin increased and decreased significantly, respectively (Fig 4), indicating the greater damage to PSII in Baijinpao than in Kangnaixin (Fig 4A, 4B).

The ϕ_Po_, ϕ_Eo_, Ψ_Eo_, and ϕ_Do_ parameters are related to the energy allocation ratio. The ϕ_Po_ indicates the PSII RC’s capacity to absorb photons and then collect energy [49]. The significant decreases in ϕ_Eo_ and Ψ_Eo_ indicate that Al stress inhibited the transfer of photosynthetic electrons beyond Q_A_ (Fig 5) [50]. Additionally, ABS/CS_m_, TR_o_/CS_m_, ET_o_/CS_m_, and DI_o_/CS_m_ are associated with energy distribution per unit cross-sectional area and RC density [32, 51]. As the Al stress treatment proceeded, ABS/CS_m_, TR_o_/CS_m_, and ET_o_/CS_m_ decreased significantly in Baijinpao. However, a significant increase in DI_o_/CS_m_ was detected for both rhododendron cultivars (Fig 6). Earlier studies indicated that stress-induced decreases in ABS/CS_m_, TR_o_/CS_m_, and ET_o_/CS_m_ may be associated with the degradation or deactivation of RCs, possibly as part of a mechanism protecting plants from stress [52, 53]. The RC-associated activation of the defense mechanism that leads to the dissipation of excess excitation energy in leaves in a timely manner, which is indicated by an increase in DI_o_/CS_m_, helps to minimize the damage to plants [48]. A recent study confirmed δR_o_ is a semi-quantitative index useful for determining the relative change in PSI [14]. In this study, δR_o_ decreased significantly in response to Al stress treatment of the two rhododendron cultivars, implying the exposure to Al stress destroys photochemical activity of PSI. Furthermore, PI_total_ has been used to indicate the overall activity of PSII, PSI, and the intersystem electron transport chain, whereas PI_ABS_ has been used to reflect the functional activity of PSII based on ABS absorption [54, 55]. In this study, both PI_total_ and PI_ABS_ decreased significantly, and the decrease Baijinpao was more significant than Kangnaixin, indicating that Al stress inhibited the overall activity of PSII, PSI, and the intersystem electron transport chain more in Baijinpao than in Kangnaixin (Fig 7).

Changes in MR_820nm_ represent alterations to the redox state of PC and P_700_ [36, 56, 57]. Therefore, measuring MR_820nm_ signals can fill in some of the “blind spots” associated with PF measurements [26, 56]. The MR_820nm_ analysis indicated that in response to an increase in the duration of the Al stress treatment, the later the lowest point of the MR/MR_0_ curve for the two rhododendron cultivars occurred, the V_ox_ increased, and the V_red_ decreased. V_ox_ can be used to evaluate the activity of PSI, V_red_ can be used to evaluate the cyclic electron transport activity [58]. Under Al stress, the increase in V_ox_ meant that the oxidation rate of PC and P_700_ increased. The decrease in V_red_ meant that the re-reduction rates of P_700_^+^ and PC^+^ decreased, which was related to the suppression of PSI, possibly because an insufficient number of electrons were transferred to PSI during the Al stress treatment [33, 36]. In addition, after Al stress, compared with Kangnaixin, the V_ox_ of Baijinpao had no significantly change, while the V_red_ of Baijinpao was significantly decreased (Fig 8). This showed that compared with Kangnaixin, the oxidation rate of PC and P_700_ of Baijinpao was not much different, while the re-reduction rate of PC^+^ and P_700_^+^ of Baijinpao is slower. Thus, under Al stress, the cyclic electron transport activity of Kangnaixin was higher and showed faster cyclic electron transport around PSI. High cyclic electron transport activity can protects PSII from excess light by producing ΔpH, which is necessary to form non-photochemical quenching [59], and may also maintain the Calvin cycle by balancing ATP/NADPH [60]. These may be the reasons why Kangnaixin has a stronger Al tolerance.

The DF induction kinetics of the two rhododendron cultivars were significantly affected by Al stress (Fig 9). The I1 point of DF is related to the increase of the transmembrane potential of the thylakoid membrane and the accumulation of the luminescent group Z^+^P_680_Q_A_Q_B_^−^ caused by PSI oxidation [61, 62]. The appearance of I2 is related to the accumulation of Z^+^P_680_Q_A_^−^Q_B_^−^ during the reduction of PQ [63]. The I1/I2 ratio is related to the electron transport capacity of the donor side of PSII [14, 64]. (I1−D2) /D2 changes are similar to I1/I2, which reflects the electron transfer rate on the acceptor side of PSII [63, 64]. In the current study, I1 and I1/I2 of both cultivars decreased significantly, indicating the decrease in the electron transport capacity of the PSII donor side. Additionally, the significant decrease in the (I1 − D2)/D2 value may be related to the loss of PSII activity and the functional impairment of PSI. The changes of curve and the decreases of parameters in the DF kinetic were more obvious for Baijinpao than for Kangnaixin, suggesting the electron transport in Baijinpao than in Kangnaixin under Al stress conditions was more severely damaged.

In conclusion, in this study, the Pn and Gs rhododendron leaves were decreased and Ci was increased under Al stress, indicating that the photosynthetic performance of leaves was decreased by non-stomatal factors. Changes in the L-band and K-band and decreases in (I1−D2) /D2 and I1/I2 indicated that the electron transfer rate on the PSII acceptor side was inhibited. The δRo was significantly decreased, which indicated that the photochemical activity of PSI was destroyed by Al stress. The results of MR_820_ showed that under Al stress, the oxidation rate of PC and P_700_ increased, and the re-reduction rate of P_700_^+^ and PC^+^ decreased. Compared with Kangnaixin, the Pn of Baijinpao decreased more significantly, the donor-side electron transfer efficiency was inhibited more seriously, and the overall functional activity of PSII, PSI and intersystem electron transfer chain was damaged more seriously under Al stress. Therefore, Kangnaixin performed better than Baijinpao under Al stress and had a higher tolerance to Al.

## Supporting information

S1 Table Rhododendron cultivars used in experiments. (XLSX)
